# Base-rate sensitivity through implicit learning

**DOI:** 10.1371/journal.pone.0179256

**Published:** 2017-06-20

**Authors:** Andrew J. Wismer, Corey J. Bohil

**Affiliations:** Department of Psychology, University of Central Florida, Orlando, Florida, United States of America; Waseda University, JAPAN

## Abstract

Two experiments assessed the contributions of implicit and explicit learning to base-rate sensitivity. Using a factorial design that included both implicit and explicit learning disruptions, we tested the hypothesis that implicit learning underlies base-rate sensitivity from experience (and that explicit learning contributes comparatively little). Participants learned to classify two categories of simple stimuli (bar graph heights) presented in a 3:1 base-rate ratio. Participants learned either from “observational” training to disrupt implicit learning or “response” training which supports implicit learning. Category label feedback on each trial was followed either immediately or after a 2.5 second delay by onset of a working memory task intended to disrupt explicit reasoning about category membership feedback. Decision criterion values were significantly larger following response training, suggesting that implicit learning underlies base-rate sensitivity. Disrupting explicit processing had no effect on base-rate learning as long as implicit learning was supported. These results suggest base-rate sensitivity develops from experience primarily through implicit learning, consistent with separate learning systems accounts of categorization.

## Introduction

Classification judgments are commonly based on observable stimulus attributes, such as the presence or absence of symptoms in a medical diagnosis. When observable traits are inconclusive regarding category membership, though, classification can be guided by other knowledge. A widely-studied example is category base rates (relative prevalence of each candidate category). Swollen glands, for example, might suggest a number of possible conditions (e.g., allergies, mononucleosis, HIV). It often makes sense on initial diagnosis to presume the presence of a common ailment. Achieving optimal classification performance (e.g., that maximizes reward or accuracy over the long run) requires combining knowledge of category base-rates (prior probabilities) with likelihood of membership in each category being considered.

Much research shows that base-rate information is often underused in classification judgments (although not entirely neglected [1]). In many studies participants receive a written summary of base-rates along with stimuli to classify, and proceed to make decisions exhibiting limited adherence to the supplied probabilities. Base-rate studies often focus on the format of the summary information (e.g., relative frequency vs. probabilities) or other contextual details to account for base-rate effects [2].

In contrast to base-rates presented in summary numeric form, several studies show that participants show sensitivity to base-rates when encountered via direct experience over a series of decision or classification trials (e.g., [3]). Sensitivity develops even though base-rate information is never explicitly communicated [4–9].

In this paradigm, base-rate influence is often gauged in terms of placement of a classification criterion or boundary (e.g., the β value in signal detection theory [10–13]). The optimal (e.g., reward-maximizing) value of β is a likelihood ratio reflecting category base-rates (and payoffs, which are not considered here). For example, if categories are presented with equal frequency (a 1:1 base-rate ratio), the optimal β value is 1. If category 1 is presented twice as frequently as category 2, the optimal β value is 2 (a 2:1 base-rate ratio), and so on. Participants typically adopt a criterion biased in the optimal direction—one that increases accuracy/reward on the high base-rate category—but not to the optimal extent. This failure to adjust sufficiently is termed “conservative cutoff placement”.

Decision criterion placement is a useful measure of base-rate sensitivity because a) performance can readily be compared to an “optimal” classifier that has knowledge of category base-rates and maximizes long-run performance, and b) base-rate sensitivity can be examined in relation to other important factors like category discriminability (*d’*) and the payoffs (benefits and costs) for correct and incorrect responses [14].

### Implicit base-rate learning

The apparent difficulty in utilizing explicit base-rate summaries, along with the fact that base-rate sensitivity can develop from direct experience, may have consequences for training in domains where classification is uncertain (e.g., radiology, dermatology). It would be beneficial to understand how base-rate sensitivity develops from experience.

Some researchers have hypothesized that base-rate sensitivity from direct experience develops through implicit learning [2, 15, 16]. Written base-rate summaries rely on conscious awareness and explicit reasoning, but sensitivity gained from experience may develop associatively over time without ever resulting in explicit awareness of relative base-rate probabilities. This hypothesis has received very little empirical investigation from researchers, although Bohil & Wismer [17] recently reported evidence suggesting that implicit learning contributes to base-rate sensitivity.

### Multiple learning systems

Although direct experience is known to improve base-rate sensitivity, direct experience is not synonymous with implicit learning. In recent years categorization researchers have posited a role for several distinct learning systems, which may be employed under different circumstances [18–20]. Many of these ideas have been supported by neuroscience results [18, 21, 22].

One influential idea in categorization research is that separate brain systems—one for explicit (verbalizable) learning and one for implicit (nonverbalizable) learning—are involved in category rule learning. This argument is the basis for the neuropsychological theory of categorization called COVIS (Competition between Verbal and Implicit Systems [23]). COVIS posits that category rule learning can be accomplished by either a prefrontal-cortex mediated explicit reasoning system or via gradual associative learning mediated by the basal ganglia. The systems compete on each learning trial to determine the categorization response. Over many trials, one system comes to dominate performance. If the explicit system is able to discover a verbalizable rule—through conscious, “explicit” hypothesis testing from trial-to-trial—that achieves high response accuracy, then this system will determine most responses. If this system fails to identify an effective verbalizable rule, though, excellent performance may still be achieved via gradual associative learning by the implicit system. Learning in the implicit system is facilitated by making a motor response on each learning trial, helping to associate category feedback with responses.

Many studies have dissociated explicit and implicit category learning behaviorally as well as in neuroimaging data (e.g., [24]). In these studies, categories are learned through a long series of training trials (i.e., through direct experience with category examples). The implication is that even though category learning takes place over many trials, learning may be mediated by either an explicit or implicit process depending on the brain system experiencing the most success. Thus, direct experience learning does not necessarily equate to implicit learning with respect to base-rate influence. Therefore, the hypothesis that implicit learning underlies base-rate sensitivity from direct experience requires testing. Although other theories of categorization exist, the current research was inspired by COVIS predictions since they directly relate to our goal of disentangling implicit learning and direct experience.

### Testing implicit and explicit learning contributions to base-rate sensitivity

We predict that base-rate learning through classification experience *does indeed* rely on implicit learning. This is because people have been shown in many categorization studies to be influenced by base-rates without explicit mention of them, and also due to the well-documented difficulty people have with explicitly presented base-rate information. Consequently, base-rate sensitivity gained from experience may or may not benefit from explicit reasoning (at least for category learning). The current research evaluates these two possibilities.

Bohil & Wismer [17] recently found support for the hypothesis that implicit learning is important for developing category base-rate sensitivity. They replicated an earlier classification experiment that disrupted implicit learning by use of an observational category training task (participants merely watched stimuli and category labels on training trials as opposed to the more standard design where each stimulus presentation is followed by a response and then feedback about category membership; following [25, 26]). Ashby and colleagues [25] showed that learning of an easily verbalized category rule—thought to be learned explicitly via conscious, trial-by-trial hypothesis testing—was not diminished by observational training, but learning of a nonverbalizable rule—thought to rely on implicit learning—was substantially disrupted. In a second experiment, Bohil & Wismer [17] disrupted implicit learning in a response-training condition by inserting a short delay (2.5 seconds) between response and feedback presentation (similar to a category rule-learning study conducted by Maddox, Ashby, & Bohil, [27]). Both methods of disrupting implicit learning—observational training and response-training with delayed feedback—led to greater conservatism in decision criterion placement (i.e., less response bias toward the high base-rate category).

Bohil and Wismer’s [17] results align with the argument that implicit learning contributes to base-rate acquisition from experience. Their studies did not, however, attempt to control the role explicit reasoning may play in base-rate learning from experience. In the current study, we factorially combined conditions in which implicit and explicit learning are supported or disrupted. Participants completed either a *response training condition* (in which implicit learning should proceed normally) or an *observational training condition* (which disrupts implicit learning). Participants also completed a secondary working memory task allowing either a *short or long* duration to process classification feedback (depending on condition). This task—a variant of the widely used Sternberg [28] working memory task—was intended to interfere with feedback processing and has been shown in several previous studies to disrupt explicit learning of a categorization rule [29–32].

We predicted that base-rate sensitivity would develop normally in the response training conditions and be limited in the observational training conditions (as indicated by more conservative decision criterion placement). We also predicted that with response training (i.e., when implicit learning is supported) disrupting the explicit system should have little effect on base-rate acquisition. This would further support the conclusion that base-rate sensitivity from experience relies on implicit learning, while explicit reasoning about base-rates has comparatively less value.

## Experiment 1

Experiment 1 tested the contribution of explicit and implicit learning processes to development of base-rate sensitivity in classification. We replicated and extended the design of Experiment 1 from Bohil & Wismer [17]. Participants learned two categories presented with unequal category base-rates and either provided a keyboard response on each trial (response training) or simply observed stimuli and category labels during training (observational training). This training manipulation varies the involvement of the implicit learning system. We expected to find larger (closer to optimal) decision criterion values in response training conditions where implicit learning was supported, and smaller criterion values (i.e., unbiased) in observational conditions where implicit learning was disrupted.

During classification training, participants also completed a secondary working memory task designed to moderate performance of the explicit learning system. Participants experienced (between subjects) either a short- or long-delay between classification feedback offset and secondary task onset on each trial. Immediate secondary task onset should disrupt working memory processing of the feedback and thus the explicit learning system, making apparent whether explicit learning contributes to base-rate sensitivity from experience. A long delay—which doesn’t interfere with feedback processing—should allow normal operation of the explicit learning system. We predicted that a short feedback processing time would not diminish criterion values in the response training conditions if explicit learning is of little help when procedural learning is intact. And if explicit learning contributes little to base-rate sensitivity, then a long delay may not improve performance in observational training conditions where procedural learning is disrupted.

### Method

#### Experimental design

Four experimental conditions—resulting from factorial combination of 2 training types (response, observational) and 2 feedback processing times (short, long)–were run in a between-subjects design. As in prior studies, all participants began by completing an unbiased base-rate “baseline” training phase to separate category structure learning from the effect of base-rates on decision criterion placement [11, 17, 33].

**Fig 1:**
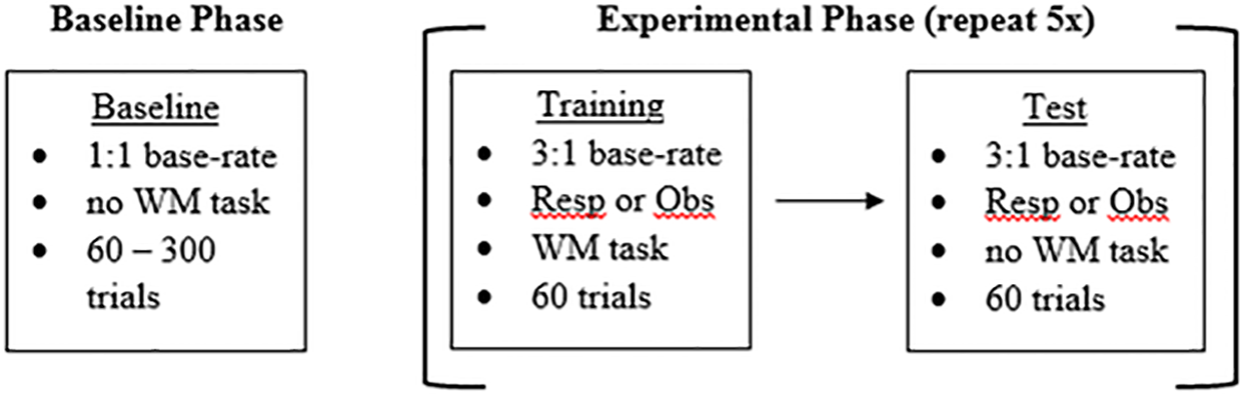
Experimental design. Participants completed the baseline phase followed by 5 cycles of alternating training and test blocks with unequal base-rates. (WM = Working memory task, Resp = response training condition, Obs = observational training condition).

After completing the baseline phase, participants completed several classification trials with unequal category base-rates. This unequal base-rate phase alternated between training blocks with trial-by-trial response feedback and test blocks without feedback. For this phase, stimuli were sampled from the two categories to produce a 3:1 base-rate ratio (i.e., Category A stimuli were presented three times as often as Category B stimuli). During this phase, participants completed five cycles of alternating 60-trial training (response or observational classification with feedback and working memory task) and test blocks (response classification without feedback and no working memory task; see Fig 1). These values were chosen to replicate several earlier studies in this area (e.g., [4, 8, 9, 33]).

The only differences among the four experimental conditions were the training phases in which one of two training types (response or observational) and one of two feedback processing times (short or long) were experienced. Category discriminability was constant across all phases (details below). The entire experiment took participants between approximately 1 to 1.5 hours to complete.

#### Participants

We conducted a power analysis with G*Power 3.1 [34] using the effect size from a study similar to the current one (Bohil & Wismer [17]; Experiment 1). The recommended sample size was 82 based on statistical test ANOVA: Fixed effects, special, main effects and interactions; alpha = .05, power = .8, effect size = .314, # groups = 2). We recruited 84 University of Central Florida undergraduate student participants. Twenty-one participants completed each condition (response-short, response-long, observational-short, and observational-long) in exchange for course credit. The study protocol was approved by the University of Central Florida Institutional Review Board, and written informed consent was provided by all participants.

#### Stimuli

Perceptual categorization stimuli were used in every phase, while working memory stimuli were only presented during the five training phases as a secondary task.

Categorization **s**timuli appeared, one per trial, as white bar graphs on a black background on a high resolution computer monitor. Bar height varied from trial-to-trial and was the indicator of category membership (again, details replicate those from several previous studies). Each bar graph was 40-pixels wide and rested on a 60-pixel wide base that was centered onscreen. Bar height ranged from about 2–6 degrees of visual angle.

Two categories—A and B—were sampled from overlapping univariate normal distributions (*Mu A* = 99.5, *Mu B* = 120.5, *SD* = 21; *d’ [category discriminability]* = 1). A set of 60 bar-height values was sampled to reflect a 3:1 base-rate ratio for training and test phases: 45 from Category A and 15 from Category B. The sample characteristics were very close to the population parameters (*Mu A* = 99.57; *Mu B* = 120.55; *SD* = 21.00). For the baseline phase (1:1 base-rate), 30 stimulus values were sampled from each category distribution (60 total), with sample values again closely matching the population characteristics (*Mu A* = 99.56; *Mu B* = 120.55; *SD* = 21.00). The presentation order for the 60 stimuli was randomized for each block but was identical for each participant.

Verbal working memory stimuli were used only in the unequal base-rate training phases (not during baseline training). Following each classification response, the working memory stimulus set (four integers, from 1–9, randomly sampled without replacement on each trial) was displayed in a row at the center of the screen horizontally spanning approximately four degrees of visual angle. The memory set was later replaced by a single integer “memory probe”. On each trial, there was a 50% chance the memory probe was a digit that appeared in the memory set. The characteristics of the memory scanning task were chosen to match those of Zeithamova & Maddox [32] (see also [29]). In these earlier studies, the secondary working-memory task was shown to disrupt learning of an easily verbalized selective attention rule that produces high accuracy. The task as implemented here should at minimum occupy working memory and somewhat diminish the effectiveness of explicit reasoning processes.

#### Procedure

**Fig 2:**
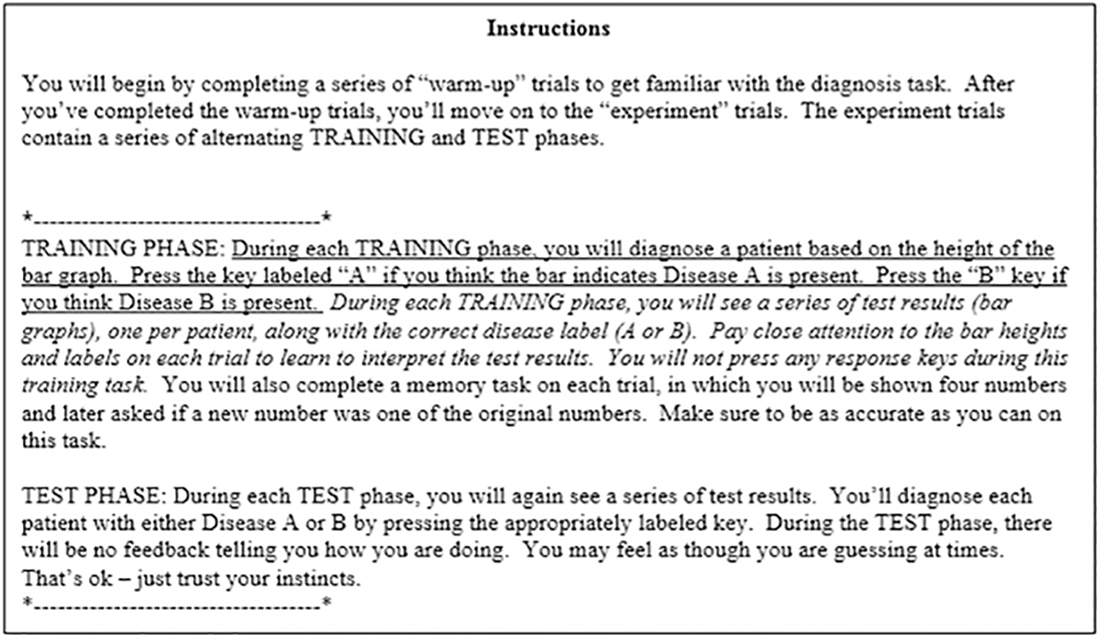
Participant instructions describing each phase of the experiment. Underlined text was seen only in response conditions, italicized text in observational conditions. All other text was seen by all participants.

Participants were informed they would complete a simulated medical diagnosis task. On each trial, a bar graph was displayed representing a hypothetical patient’s test result. The test result (i.e., bar graph) would be used to distinguish between two diseases—A and B. Participants were told they would start out by guessing but improve over time. They were also informed perfect performance was impossible because the test was an imperfect predictor, but over time they should be able to achieve a high level of performance. The baseline phase was described as a series of warm-up trials to become familiar with the task. They were told that after completing the warm-up trials, they would move on to the experiment trials consisting of alternating training and test phases. Participants were given identical instructions in all conditions with the exception of the training phase description, which differed for response and observational conditions (see Fig 2).

During the baseline phase (regardless of training type), participants viewed each categorization stimulus for 500ms above a line of text saying "Diagnose the patient as having disease A or B". The stimulus was then removed from the screen (with the text remaining), allowing for participant responses. They responded with ‘A’ or ‘B’ by pressing a keyboard button (key ‘z’ or ‘m’ labeled as A and B). They had a maximum of 10 seconds to respond, although participants responded much faster (average observed response time was about a half second in all conditions. We don’t consider response times further). To provide corrective feedback, the category label was then displayed (500ms), followed by a 2000ms inter-trial interval (ITI). The baseline phase had equal category base-rates (30 of each category presented for each 60-trial baseline block) and did not include the secondary working memory task.

Participants completed a minimum of 60 baseline trials (i.e., all 30 stimuli from each category), and up to a maximum of 300 trials. After the first 60 trials, accuracy for the most recent 60 trials was calculated (and recalculated every 10 trials thereafter). If accuracy reached 65% or higher (optimal accuracy during baseline phase was 69%), the baseline phase ended and the participant moved on to the biased (3:1) base-rate training trials (where optimal accuracy was 78%). This criterion ensured participants had learned the categories well, allowing participants to proceed to experimental trials with only three more incorrect responses than the optimal classifier for the most recent 60 trials. Participants failing to reach this criterion in 300 trials continued on to complete the rest of the experiment (see Results for additional consideration of baseline performance).

The training blocks were the only aspect of the experiment that differed among conditions. The category base-rate ratio (3:1) was the same for all participants. However, the training type and onset delay of the secondary task differed across conditions during the training blocks. In response training conditions, participants guessed either category A or B by pressing the appropriate keyboard button. In observational training conditions no response was made. Instead, stimuli and category labels were merely observed on each trial. The stimulus timings for the response condition were as follows: 1000ms stimulus on screen with text "Diagnose the patient as having disease A or B", removal of stimulus and pause for participant response (max 10 seconds), followed by a blank screen (500ms) and 500ms feedback display. In the observational conditions, the stimulus was shown for 1000ms, followed by a blank screen for 500ms, and then the category label for 500ms.

**Fig 3:**
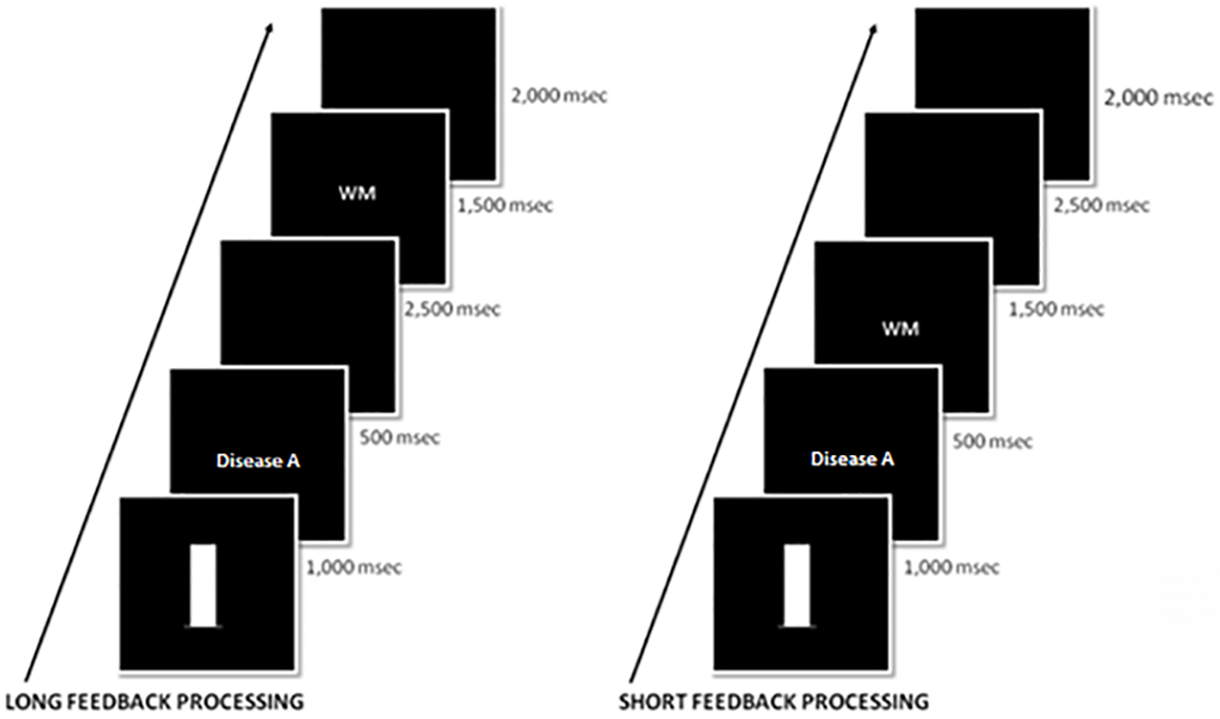
Layout of trials in the training phase for long and short feedback processing time response conditions. Layout was identical for response and observational conditions, with the exception that observational conditions were shown the category label without making a response.

During training trials, category label offset was followed by the verbal working memory task. In the short feedback processing conditions, the working memory task began immediately following the offset of the category label. Fig 3 illustrates event timing for the long and short delay conditions. In the long feedback processing conditions, the working memory task began 2500ms after category label offset. The sequence of the working memory task was as follows (block labeled "WM" in Fig 3): presentation of the 4-digit working memory stimulus set (500ms), then a blank screen (1000ms), followed by presentation of the 1-digit memory probe (response-terminated). Following a response to the working memory probe (keys 'a' or 'l' labeled as ‘Yes’ and ‘No’), an ITI of 2000ms occurred. No feedback on the working memory task was given, but in-between blocks participants were encouraged to keep their accuracy on the working memory task high. In short conditions, the ITI was preceded by an additional blank screen of 2500ms to counterbalance the interval occurring between category label and working memory task in long conditions.

During test blocks—regardless of training condition—participants viewed a stimulus (1000ms) above the words "Diagnose the patient as having disease A or B", and the stimulus was then removed allowing for participant classification response (max 10 seconds). Instead of receiving feedback, participants were shown the message “your response has been recorded” (500ms) after responding. The same ITI of 2000ms was used. During training and test blocks with unequal base-rates, optimal accuracy was 78%.

### Results

Most participants (90%) completed the baseline phase in fewer than 300 trials (76 of 84 participants; *Mdn* = 110 trials), and the median number of trials to reach criterion did not differ among experimental conditions (*p* = .968). A regression analysis indicated that the number of baseline trials completed did not significantly affect one's decision criterion in the unequal base-rate phase [e.g., Block 4: *F*(1, 80) = .04, *p* = .850; Block 5: *F*(1, 82) = 0.48, *p* = .493]. By the time participants began the experimental (unequal base-rate) phase, their classification accuracy was in most cases within three errors of the optimal classifier over the most recent 60 trials. Thus, the results reported below are based on the full set of 84 participants (none were omitted based on their baseline phase performance).

#### Accuracy

Table 1 displays average proportion of correct classification responses across blocks for Experiment 1. We conducted a mixed-factor ANOVA using accuracy as the dependent measure (between: 2 training types X 2 feedback processing times; within: 5 test blocks). Following the convention of Dienes [35] and Jeffreys [36], we also report Bayes Factors (BF) which help to distinguish between results supporting the null hypothesis and those resulting from a lack of statistical power. We interpret BF > 3 as evidence for the experimental hypothesis, BF < .33 as evidence for the null hypothesis, and values in between as equivocal. Data from two participants were omitted because their accuracy value for at least one block was more than three standard deviations from the mean and so were considered outliers (one each in the response-long and observational-short conditions).

**Table 1:** Average proportion correct (& signal-detection β values) by condition & block in Experiment 1.

Condition	Test block 1	Test block 2	Test block 3	Test block 4	Test block 5
Individual (*n* = 21/cond)					
Response-Short	.62 (1.02)	.66 (1.25)	.68 (1.28)	.65 (1.32)	.69 (1.66)
Response-Long	.63 (1.04)	.71 (1.31)	.69 (1.37)	.70 (1.39)	.72 (1.73)
Observational-Short	.61 (1.01)	.66 (1.06)	.61 (1.08)	.65 (1.12)	.67 (1.29)
Observational-Long	.63 (1.08)	.64 (1.20)	.67 (1.26)	.63 (1.20)	.62 (1.16)
Average (*n* = 42/cond)					
Response	.62 (1.03)	.68 (1.28)	.68 (1.33)	.67 (1.35)	.70 (1.70)
Observational	.62 (1.04)	.65 (1.13)	.64 (1.17)	.64 (1.16)	.64 (1.23)
	# (#)	* (*)	* (#)	# (*)	** (**)

*Note*. Observational = observational-training; Response = response-training; Short = short feedback processing time; Long = long feedback processing time. *Note*. Significance of β comparisons across training conditions is reported below proportions for average training conditions (# *p* ≥ .05; * *p* < .05; ** *p* < .01).

There was a main effect of block (Greenhouse-Geisser corrected), *F*(3.18, 248.30) = 7.29, *p* < .001, η_p_^2^ = .085, with accuracy increasing over blocks. There was a main effect of training type on accuracy, *F*(1, 78) = 4.64, *p* = .034, η_p_^2^ = .056, with higher average accuracy in response conditions (*M* = .67) than in observational conditions (*M* = .64), but no main effect of feedback processing time, *F*(1, 78) = 0.59, *p* = .443, η_p_^2^ = .007. The interaction of block and training type was marginally significant, *F*(3.18, 248.30) = 2.59, *p* = .05, η_p_^2^ = .032, as accuracy increased more across blocks in the Response conditions than in the Observation conditions. The interaction of block, training type, and working memory condition was also significant, *F*(3.18, 248.30) = 2.65, *p* = .046, η_p_^2^ = .033. All other interactions were non-significant (*p’s* > .225).

Bayesian analyses were conducted for each dependent variable using JASP computer software [39]. Bayesian analyses are reported following the example of Wagenmakers et al. [37] and Wagenmakers et al. [38]. A Bayesian analysis of the accuracy data revealed overwhelming support for including an effect of block (*BF*_*inclusion*_ = 307.73), while all other variables received little-to-no support for including an effect (all *BF*_*inclusion*_’s < 1.27). The model that received the most support against the Null model is the two main effects model, block + training type (*BF*_*10*_ = 976.16). Adding the main effect of feedback processing time decreases the degree of this support by a factor of 976.16/284.98 = 3.43. This is the Bayes factor in favor of the two main effects model versus the three main effects model. Adding the interaction of training type and feedback processing time decreases the degree of support by a factor of 976.16/145.54 = 6.71.

As a manipulation check, we ran a mixed factor ANOVA on accuracies from the working memory task (in the training blocks) to verify that accuracy on the secondary task did not differ between training conditions. Critically, there was no difference between response (*M* = .95) and observational (*M* = .94) conditions (*p* = .676).

#### Signal detection

Table 1 displays average signal detection criterion values (β) across blocks for Experiment 1 (see values inside parentheses). Correct responses for stimuli from the high base-rate category were considered “hits”, and incorrect responses to stimuli from the low base-rate category were considered “false alarms”. Response bias (β) measures were derived from these. Data from four participants were omitted because their β value for at least one block was more than three standard deviations from the mean and so were considered outliers (two in the response-short, one in the response-long, and one in observational-long conditions). With β as a dependent measure, we performed a mixed-factor ANOVA to compare all four experimental conditions (between: 2 training types X 2 feedback processing times; within: 5 test blocks).

**Fig 4:**
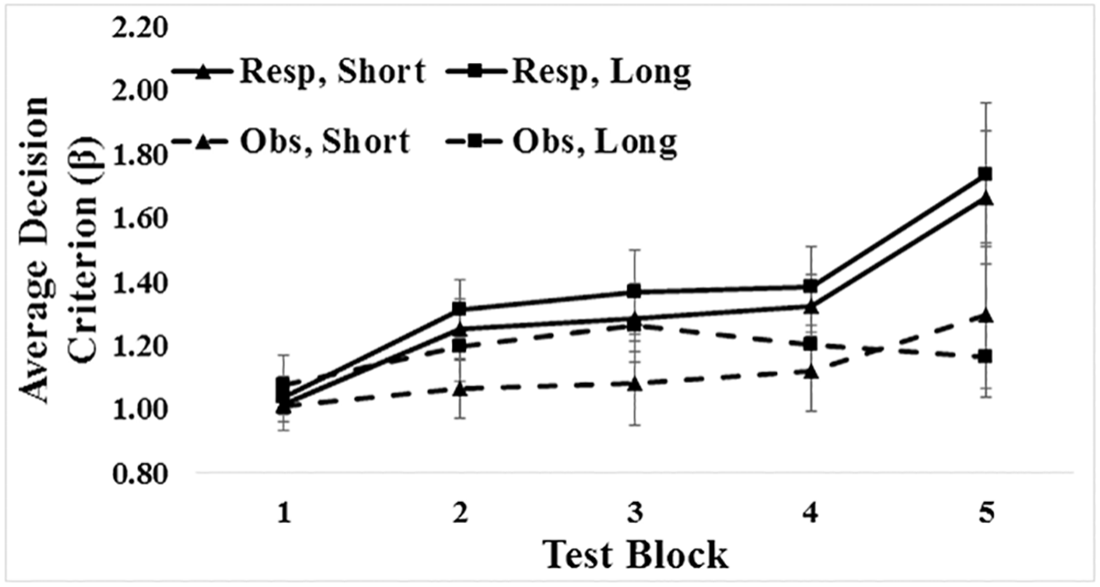
Average signal detection β values by condition. Resp = response training; Obs = observational training; Short = short feedback processing time; Long = long feedback processing time. Optimal β value = 3. Error bars show standard error.

There was a main effect of training type, *F*(1, 76) = 4.45, *p* = .038, η_p_^2^ = .055, with larger β values in response (*M* = 1.34) than in observational (*M* = 1.15) conditions (See Fig 4). There was also a main effect of block. Values of β increased toward optimal (β_optimal_ = 3) across blocks, *F*(2.35, 178.39) = 10.05, *p* < .001, η_p_^2^ = .117. There was no main effect of feedback processing time, *F*(1, 76) = .42, *p* = .519, η_p_^2^ = .005. There was an interaction between block and training type, *F*(2.35, 178.39) = 3.76, *p* = .019, η_p_^2^ = .047, such that β's in response conditions increased more rapidly than β's in observational conditions. No other interactions were significant (all *p*'s > .322).

A Bayesian analysis of the signal detection criterion data revealed overwhelming support for including an effect of block (*BF*_*inclusion*_ = 64824.53) as well as support for including effects of training type (*BF*_*inclusion*_ = 3.34) and block * training type (*BF*_*inclusion*_ = 6.25). All other variables received support against having an effect (all *BF*_*inclusion*_’s < .136). The model that received the most support against the Null model is the model with two main effects and one interaction, block + training type + block * training type (*BF*_*10*_ = 314005.8). Adding the main effect of feedback processing time decreases the degree of this support by a factor of 314005.80/85517.83 = 3.67. This is the Bayes factor in favor of the two main effects and one interaction model versus the three main effects and one interaction model. Adding the interaction of training type and feedback processing time decreases the degree of this support by 314005.80/26439.02 = 11.88.

We also ran two separate ANOVAs looking at the effect of the working memory manipulation on response conditions and observational conditions alone. There was no main effect of feedback processing time on response conditions (*p* = .662) or observational conditions (*p* = .635).

#### Modeling

In order to better understand the response strategy used by each participant, we fit three decision-bound models to each data set (separately for each participant and for each block). The models assume either a decision criterion value (β) that is fixed at a value to instantiate different hypotheses (e.g., sensitivity or neglect of base-rates), or estimate a decision criterion value that best describes the participant’s classification responses to the stimuli presented during the experiment (a detailed description of decision-bound models can be found elsewhere; e.g., [40]). The location of the bound in stimulus space is a reflection of the participant’s response bias toward either category. For example, a decision bound equidistant between the category means would reflect complete insensitivity to base-rates (e.g., β = 1). Decision-bound models have informed our interpretation of base-rate learning from experience in several previous studies, and they allow us to test specific hypotheses based on statistical comparisons at the individual participant level [4, 11, 17, 41].

We compared three models: (1) an unbiased-boundary model, (2) an optimal model, and (3) a free-boundary model. The first two models each include only one free parameter: a noise parameter which estimates a combination of perceptual and criterial noise (i.e., variation in stimulus perception over time and imperfect memory for criterion location over trials, respectively). The unbiased-boundary model fits the data using a decision criterion fixed at an unbiased value (β = 1), and the optimal model fits the data using a decision criterion fixed at the optimal value (β = 3). In other words, the unbiased model tests the assumption that the participant was not sensitive to the base-rate manipulation at all, and the optimal model tests the assumption that the participant used the optimal (long-run reward maximizing) bound. The free-boundary model included two free parameters: a decision-bound parameter and the noise parameter. The model was free to estimate the criterion value that best describes the participant’s classification responses, separately for each block of test trials. For each model, maximum likelihood estimation was used to determine best-fitting parameter values for each data set (i.e., each model predicted the probability of responding category 1 or 2 on each trial given each value of the decision bound, and free parameters were adjusted to bring model predictions as close as possible to the observed responses). All models included the noise parameter. The decision criterion value was fixed for the unbiased and optimal models (at the values described above), and the decision criterion was an additional free parameter in the free-boundary model. For each subject and block, the most parsimonious description of each participant’s responding was determined based on model fit comparisons using Akaike Weights [42].

Table 2 displays the proportion of cases for which each model provided the most parsimonious account of an individual's data, as well as the β value estimated by the free-boundary model. The free-boundary model and the optimal model were often more parsimonious than the unbiased-boundary model, indicating that in many cases decision criterion values reflected sensitivity to unequal base-rates (i.e., biased in favor of the high base-rate category). Participants in response conditions were more likely to be best described by the optimal model than in observational conditions. This difference between training types approached significance (*z* = 1.546, *p* = .06; collapsed over blocks).

**Table 2:** Proportion of participants (and FRB model β values) in Experiment 1 whose data were most parsimoniously accounted for by each model.

Condition		Test block 1	Test block 2	Test block 3	Test block 4	Test block 5
Individual (*n* = 21/cond)						
Resp-Short	UNB	0.19 (0.74)	0.14 (0.9)	0.14 (1.26)	0.14 (0.6)	0.05 (1.39)
	OPT	0.33 (3.17)	0.57 (3.55)	0.38 (3.47)	0.57 (4.61)	0.67 (4.13)
	FRB	0.48 (2.32)	0.29 (4.28)	0.48 (5.31)	0.29 (3.72)	0.29 (5.8)
Resp-Long	UNB	0.48 (1.17)	0.33 (1.06)	0.29 (0.8)	0.38 (1.04)	0.19 (0.94)
	OPT	0.29 (3.28)	0.48 (3.14)	0.52 (3.56)	0.52 (3.93)	0.62 (2.92)
	FRB	0.24 (0.84)	0.19 (2.43)	0.19 (3.3)	0.1 (2.9)	0.19 (3.71)
Obs-Short	UNB	0.57 (0.98)	0.52 (0.93)	0.29 (1.02)	0.24 (1.02)	0.29 (0.91)
	OPT	0.19 (3.36)	0.43 (2.82)	0.38 (3.54)	0.38 (2.95)	0.38 (2.97)
	FRB	0.24 (0.63)	0.05 (1.49)	0.33 (1.3)	0.38 (2.18)	0.33 (2.19)
Obs-Long	UNB	0.48 (1.04)	0.48 (1.11)	0.14 (0.78)	0.43 (1.1)	0.38 (0.77)
	OPT	0.14 (3.9)	0.33 (2.96)	0.38 (2.74)	0.38 (1.83)	0.29 (2.77)
	FRB	0.38 (1.62)	0.19 (1.81)	0.48 (1.45)	0.19 (2.5)	0.33 (1.63)
Average (*n* = 42/cond)						
Response		0.33 (1.99)	0.24 (2.85)	0.21 (3.38)	0.26 (3.26)	0.12 (3.59)
Observational		0.52 (1.51)	0.50 (1.82)	0.21 (1.96)	0.33 (1.92)	0.33 (1.88)
		* (#)	** (**)	# (**)	# (**)	* (**)

*Note*. Obs = observational-training; Resp = response-training; Short = short feedback processing time; Long = long feedback processing time; UNB = unbiased boundary model; OPT = optimal boundary model; FRB = free boundary model. *Note*. Significance of comparisons across training conditions is reported below proportions for average training conditions (# *p* ≥ .05; * *p* < .05; ** *p* < .01).

The unbiased-boundary model fared better in observational conditions (*z* = 3.14, *p* < .001; collapsed over blocks) than in the response conditions. There were significantly more cases of base-rate neglect in the observational training condition than in the response training condition, particularly by the end of training (block 5). Decision bound (β) estimates from the free-boundary model were larger for response conditions than for observational conditions in each block (*p* < .05 for blocks 2–5). So the modeling results corroborate, at the individual participant level, our findings based on the aggregate signal detection analyses above.

With respect to feedback processing time, participants in the response-long condition were more often accounted for by the unbiased model than those in the response-short condition (*z* = 2.88, *p* < .005; collapsed across blocks). This was especially apparent in block 4 of the response training condition (*z* = 2.17, *p* < .05). No other delay comparisons were significant. We consider the implications of this finding in the General Discussion section.

### Discussion

Experiment 1 tested our hypothesis that base-rate sensitivity develops from classification experience when the implicit learning system is engaged (e.g., when accompanied by a motor response). This was the case in the response training conditions, where participants made a keyboard response to classify each stimulus. In the observational training conditions, participants merely observed category exemplars along with category labels during training trials. Observational training is known to disrupt category rule acquisition with two-dimensional categories when participants must learn which dimensions are relevant for classification [25]. When the stimuli are 1-dimensional as in the current study there is no question as to which dimension should be used to classify, but rather the location of the criterion must be determined. (Other recent studies have looked at criterion placement—which can be considered a sub-process of rule learning after relevant dimensions have been identified by the learner—in relation to separate learning systems, but not in the context of base-rate learning; e.g., [43]). Our hypothesis in this case is that learning the optimal criterion location in response to unequal category base-rates makes use of implicit learning.

The current data support our hypothesis. Decision criterion values—based on signal detection and decision-bound modeling analyses—were closer to optimal after response training. Criterion values in observational training conditions were significantly less biased in the optimal direction. Accuracy was also significantly higher in the response training than in the observational training conditions, although the BF value suggested insufficient test sensitivity for a definitive conclusion in Experiment 1. The current results replicate the pattern found in Bohil & Wismer’s [17] Experiment 1 but also provides insight into the relative contribution of explicit learning when base-rate sensitivity is gained from experience.

The secondary working memory task either disrupted or left intact the full potential of the explicit learning system (in the short and long feedback processing conditions, respectively). In the current study—again as judged by signal detection and decision-bound modeling analysis—disruption of the explicit learning system had no effect on criterion placement. In response conditions—when the implicit system was intact—base-rate sensitivity developed equally regardless of whether participants had a short or long period to contemplate corrective feedback. In the observational training conditions—when the implicit learning system was disrupted—there was no significant increase toward optimal in criterion values during the long-delay conditions. Similar conclusions can be drawn from the accuracy results, and BF values for accuracy and β values indicated evidence in favor of the null hypothesis with respect to feedback processing time. There seems to have been no benefit from an intact explicit learning system in the observational training conditions.

One prominent trend in the current data is a gradual increase in criterion values over blocks in the response conditions. As in many previous studies, we included the equal base-rate baseline training phase to separate category learning from the effect of the subsequent base-rate manipulation. During the baseline training phase, participants learn to use an unbiased decision criterion (i.e., β = 1). It may be the case that after switching to the experimental (unequal base-rate) trials, the effect of base-rates on criterion values was impeded somewhat by interference from this prior learning. To explore this possibility, we replicated the current study in Experiment 2, removing the initial baseline training phase.

In Experiment 2, the categories were learned with unequal base-rates. We wished to see if the difference between response and observational training might appear earlier in performance. If the baseline learning phase does interfere with the later influence of unequal base-rates, then we should see evidence of earlier criterion adjustment when the baseline training phase is omitted. If this is the case, then we might also expect to see more of an effect—if any—of the explicit learning system manipulation. Perhaps any influence of unequal base-rates over the explicit system was limited by interference from the prior baseline criterion training. Experiment 2 replicates the design of Experiment 1 but simply omits the baseline training phase.

## Experiment 2

In Experiment 1, the difference between response and observational conditions was most pronounced at the end of training although criterion values tended to be larger across blocks with response training. A plausible explanation for this gradual effect of implicit learning could be inertia carried over from the baseline category training phase. During the unequal base-rate phase of training, the participant must overcome prior learning of the decision criterion, which may somewhat inhibit learning a new criterion that reflects the asymmetric category base-rates.

In Experiment 2, we sought to replicate Experiment 1 but without this potential source of influence over criterion placement. We again compared learning in response and observational training conditions. Also, we again manipulated the feedback processing time after each categorization response by including the working memory task following a short or long delay as in Experiment 1. In Experiment 2, participants learned the base-rates and category structures simultaneously.

### Method

The design and procedure of Experiment 2 was identical to that of Experiment 1 except for the exclusion of the 1:1 base-rate baseline phase. In addition, in order to assess conclusions regarding null results for processing time, we increased our sample size to reflect power = .9. We recruited 155 University of Central Florida undergraduate student participants. There were 39, 39, 39, and 38 participants in the response-short (R-S), response-long (R-L), observational-short (O-S), and observational-long (O-L) conditions, respectively. All participants completed 600 categorization trials (300 training, 300 test as in Experiment 1), and the experiment lasted approximately one hour. Participants received course credit for completing the task. The study protocol was approved by the University of Central Florida Institutional Review Board, and written informed consent was provided by all participants.

### Results

Fifteen participants were classified as outliers and were subsequently removed list-wise from all analyses. Participants were classified as outliers if either: 1) their estimated criterion (β) in any block was more than 3 standard deviations from the mean, or 2) in the final test block, either category was selected only once or not at all. The first condition removes major outliers from the data, while the second condition guards against extreme response rates that have a disproportionate effect on beta values. The resulting sample sizes for R-S, R-L, O-S, and O-L were 35, 34, 38, and 33, respectively.

#### Accuracy

Table 3 displays average classification accuracy across blocks for Experiment 2. We conducted a mixed-factor ANOVA with classification accuracy as the dependent measure (between: 2 training types X 2 feedback processing times; within: 5 test blocks). There was a main effect of block on accuracy, *F*(3.44, 467.57) = 11.24, *p* < .001, η_p_^2^ = .076, with accuracy increasing across blocks. There was also a large main effect of training type, *F*(1, 136) = 23.30, *p* < .001, η_p_^2^ = . 146, with higher average accuracy in response conditions (*M* = .69) than in observational conditions (*M* = .62). There was again no main effect of feedback processing time [*F*(1, 136) = 0.18, *p* = .676, η_p_^2^ = .001], nor were there any significant interactions (all *p*’s > .384). Again, a manipulation check of accuracy on the working memory task in training blocks again revealed no differences between response (*M* = .94) and observational (*M* = .95) conditions (*p* = .159).

**Table 3:** Average proportion correct (& signal-detection β values) in each condition and block in Experiment 2.

Condition	Test block 1	Test block 2	Test block 3	Test block 4	Test block 5
Individual					
Resp-Short (*n* = 35)	.65 (1.25)	.69 (1.75)	.68 (1.42)	.70 (1.45)	.70 (1.59)
Resp-Long (*n* = 34)	.66 (1.24)	.68 (1.41)	.70 (1.86)	.70 (1.40)	.70 (1.35)
Obs-Short (*n* = 38)	.59 (1.05)	.66 (1.29)	.63 (1.29)	.64 (1.22)	.64 (1.13)
Obs-Long (*n* = 33)	.58 (0.91)	.63 (1.15)	.60 (1.13)	.63 (1.19)	.62 (1.30)
Average					
Response (*n* = 69)	.66 (1.25)	.69 (1.58)	.69 (1.64)	.70 (1.43)	.70 (1.47)
Observ. (*n* = 71)	.59 (0.98)	.65 (1.22)	.62 (1.21)	.64 (1.20)	.63 (1.21)
	** (**)	** (**)	** (**)	** (**)	** (**)

*Note*. Obs = observational-training; Resp = response-training; Short = short feedback processing time; Long = long feedback processing time. *Note*. Significance of β comparisons across training conditions is reported below proportions for average training conditions (** *p* < .01).

A Bayesian analysis of the accuracy data revealed overwhelming support for including effects of block (*BF*_*inclusion*_ = 552985.30) and training type (*BF*_*inclusion*_ = 1571.7), but all other variables received support against having an effect (all *BF*_*inclusion*_’s < 0.137). The model that received the most support against the Null model is the two main effects model, block + training type (*BF*_*10*_ = 6.077 * 10^9). Adding the main effect of feedback processing time decreases the degree of this support by a factor of 6.077*10^9/1.308*10^9 = 4.65. This is the Bayes factor in favor of the two main effects model versus the three main effects model. Adding the interaction of training type and feedback processing time decreases the degree of this support by a factor of 6.077*10^9/4.577*10^8 = 13.28.

#### Signal detection

**Fig 5:**
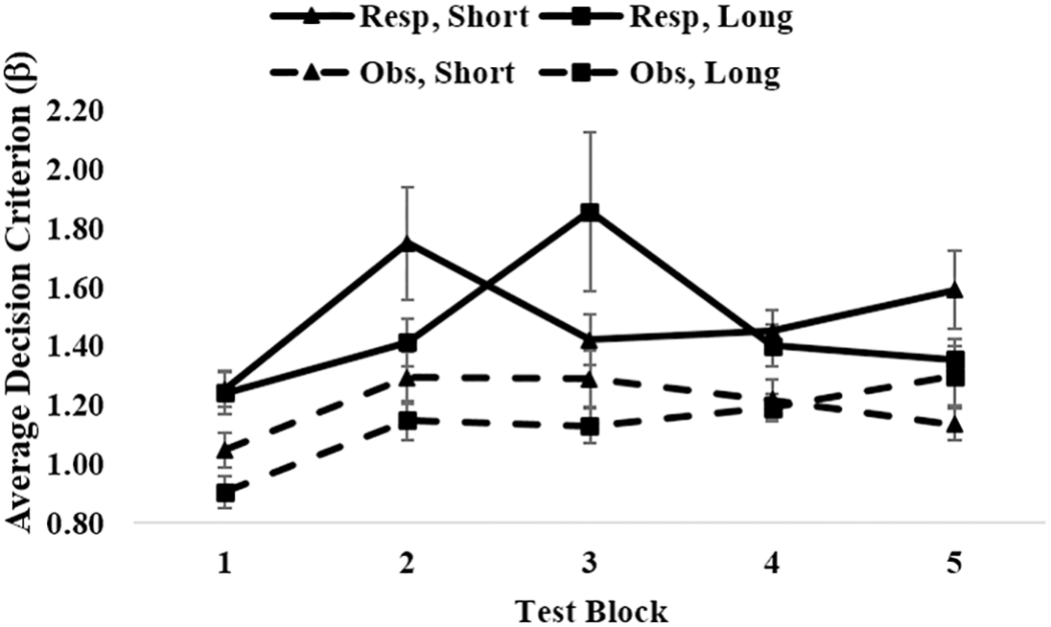
Average signal detection β values in Experiment 2 by condition. Resp = response training; Obs = observational training; Short = short feedback processing time; Long = long feedback processing time. Optimal β value = 3. Error bars represent standard error.

Fig 5 displays average signal detection criterion values (β) across blocks for Experiment 2 (see values in parentheses). We conducted the same analyses as in Experiment 1 using β as a dependent measure (Mixed factor ANOVA: between: 2 training types X 2 feedback processing times; within: 5 test blocks). There was again a large main effect of training type, *F*(1, 136) = 22.72, *p* < .001, η_p_^2^ = .143, with larger β values in response (*M* = 1.47) than observational (*M* = 1.17) conditions. There was a main effect of block, with β values increasing toward optimal (β_optimal_ = 3) across blocks, *F*(2.83, 384.42) = 7.45, *p* < .001, η_p_^2^ = .052. There was no main effect of feedback processing time, *F*(1, 136) = 0.63, *p* = .430, η_p_^2^ = .005. The interactions of block and training type, *F*(2.83, 384.42) = 0.89, *p* = .441, η_p_^2^ = .007, block by feedback processing time, *F*(2.83, 384.42) = 2.23, *p* = .088, η_p_^2^ = .016, and training type by feedback processing time, *F*(1, 136) = 0.03, *p* = .866, η_p_^2^ < .001,were not significant. There was however a significant interaction among block, training type, and feedback processing time, *F*(2.83, 384.42) = 4.33, *p* = .006, η_p_^2^ = .031.

A Bayesian analysis of the signal detection criterion values revealed overwhelming support for including effects of block (*BF*_*inclusion*_ = 282.77) and training type (*BF*_*inclusion*_ = 1040.68), but all other variables received support against having an effect (all *BF*_*inclusion*_’s < 0.10). The model that received the most support against the Null model is the two main effects model, block + training type (*BF*_*10*_ = 1961000). Adding the main effect of feedback processing time decreased the degree of this support by 1961000/351942 = 5.57. This is the Bayes factor in favor of the two main effects model versus the three main effects model. Adding the interaction of training type and feedback processing time decreases the degree of support by a factor of 1961000/66945.4 = 29.29.

We also ran two individual ANOVAs looking separately at the effect of the working memory manipulation on response and observational training conditions. There was no main effect of feedback processing time on response conditions (*p* = .717) or observational conditions (*p* = .369). However, there was some performance disruption caused by the working memory task in the observational training condition. In block 5, β values were larger in the long-delay condition than in the short delay condition for subjects receiving observational training, *F*(1, 35) = 5.538, *p* < .05).

#### Modeling

As in Experiment 1, we fit three decision-bound models to each data set (unbiased-boundary, optimal, free-boundary). Table 4 displays the proportion of cases for which each model best accounted for an individual’s data, along with decision criterion (β) values from the free-boundary model. Participants in response conditions were more likely to be best described by the optimal model than in observational conditions. This difference between training types approached significance (*z* = 1.531, *p* = .*06*; collapsed across blocks). The unbiased-boundary model fared better in observational conditions than in response conditions (*z* = 7.817, *p* < .001; collapsed across blocks). There were again more cases of base-rate neglect in the observational training condition than in the response training condition. And again the free-boundary model decision criterion (β) estimates were significantly larger in each block in response conditions than in observational conditions (*p* < .001 in each block). There was a significant effect of the delay manipulation in block 1 of the response conditions, where more participants were best fit by the unbiased model in the long-delay condition (z = 1.717, *p* < .05), similar to the modeling result in Experiment 1.

**Table 4:** Proportion of participants (and FRB model β values) in Experiment 2 whose data were most parsimoniously accounted for by each model.

Condition		Test block 1	Test block 2	Test block 3	Test block 4	Test block 5
Individual						
Resp-Short (*n* = 35)	UNB	0.15 (0.88)	0.13 (0.9)	0.1 (1.2)	0 (-)	0.03 (1.31)
	OPT	0.49 (4.33)	0.62 (3.96)	0.59 (4.21)	0.74 (3.59)	0.54 (4.25)
	FRB	0.36 (4.08)	0.26 (4.62)	0.31 (5.03)	0.26 (6.29)	0.44 (5.65)
Resp-Long (*n* = 34)	UNB	0.33 (1.05)	0.15 (1.08)	0.08 (0.7)	0.15 (1.17)	0.08 (1.07)
	OPT	0.49 (3.67)	0.62 (3.24)	0.51 (3.61)	0.56 (3.16)	0.51 (3.46)
	FRB	0.18 (2.41)	0.23 (6.17)	0.41 (6.15)	0.28 (6.77)	0.41 (5.12)
Obs-Short (*n* = 38)	UNB	0.44 (0.84)	0.31 (0.96)	0.33 (0.97)	0.31 (1.05)	0.28 (0.9)
	OPT	0.38 (3.65)	0.59 (3.82)	0.54 (3.65)	0.49 (3.74)	0.56 (3.98)
	FRB	0.18 (1.94)	0.1 (3.21)	0.13 (3.8)	0.21 (2.61)	0.15 (4.52)
Obs-Long (*n* = 33)	UNB	0.53 (0.77)	0.5 (0.89)	0.29 (0.91)	0.32 (0.7)	0.32 (0.65)
	OPT	0.29 (4.5)	0.42 (3.52)	0.37 (3.65)	0.42 (3.72)	0.37 (4.04)
	FRB	0.18 (0.78)	0.08 (2.66)	0.34 (2.42)	0.26 (2.49)	0.32 (2.98)
Average						
Response (*n* = 69)		0.24 (3.19)	0.14 (3.72)	0.09 (4.38)	0.08 (4.13)	0.05 (4.42)
Observational (*n* = 71)		0.48 (1.99)	0.40 (2.56)	0.31 (2.65)	0.31 (2.61)	0.30 (2.92)
		** (**)	** (**)	** (**)	** (**)	** (**)

*Note*. Obs = observational-training; Resp = response-training; Short = short feedback processing time; Long = long feedback processing time; UNB = unbiased boundary model; OPT = optimal boundary model; FRB = free boundary model. *Note*. Significance of comparisons across training conditions is reported below proportions for average training conditions (** *p* < .01).

### Discussion

The results of Experiment 2 replicate those from Experiment 1. In fact they seem to provide clearer support for our hypotheses. In observational training conditions, there was again relatively little bias in decision criterion values and very little change in values over blocks. In response training conditions, on the other hand, the influence of base-rates on criterion placement was clear. Response training led to larger values reflecting sensitivity to the base-rate difference between categories. Delay condition again had no main effect on performance in either response or observational training conditions, although delay condition did disrupt performance in the short- compared to long-feedback processing time conditions in the final block of trials in observational training conditions. Performance in response training conditions was not diminished by limiting feedback processing time, and performance in observational training conditions gained only limited benefit from the longer feedback processing time.

Regarding the removal of baseline training in Experiment 2, response training criterion values were clearly adjusted in the optimal direction as early as the first block of trials. This supports the argument that Experiment 1 baseline training may have interfered with later base-rate influence on criterion placement. In the observational conditions, there was no analogous increase in criterion values in the early training blocks, and very little increase as training progressed.

## General discussion

We explored the contributions of implicit and explicit learning to developing category base-rate sensitivity via direct experience in a classification task (instead of summary presentation of base-rate information). In two experiments, participants learned to classify simple perceptual stimuli (bar graph heights) sampled from two categories. Members of one category were presented three times as often as the other (i.e., in a 3:1 base-rate ratio). Both experiments combined manipulations to disrupt explicit learning, implicit learning, or both.

Learning took place through either response or observational training. In response training conditions, participants made a keyboard response to classify each stimulus, followed by corrective feedback (the correct category label). In observational training conditions, participants again viewed a category stimulus on each trial, followed by the associated category label. But they did not make any response during training trials; they merely observed stimuli and category labels. In both response and observational conditions, five training blocks (as just described) alternated with five test blocks. During each test block, participants made a keyboard response to classify each stimulus (regardless of their training condition). No category label feedback was provided during test blocks. Our analyses were based on the data from the test blocks, which provided a snapshot over time of base-rate sensitivity.

Along with classification, during training blocks participants also completed a secondary working memory task (the widely-used Sternberg memory-scanning task thought to occupy working memory resources). After feedback offset for each classification trial, the working memory task began either immediately (short delay condition) or after a few seconds (long delay condition). The short delay condition was intended to disrupt learners’ ability to consciously reason about the categorization feedback just received for that trial.

Based on much previous research, response training is thought to engage implicit learning mediated by the basal ganglia. With observational training, performance of this learning system has been shown to be disrupted (e.g., [25, 26]). If base-rate sensitivity from direct experience does rely on implicit learning, as we hypothesize, then performance should be disrupted in observational conditions, but should proceed normally in response conditions. On the other hand, the explicit learning system is thought to rely on working memory. Thus, the secondary working memory task tests influence of the explicit learning system on base-rate sensitivity from experience. If the explicit system contributes to base-rate sensitivity, then limiting feedback processing with rapid onset of the secondary task (which utilizes working memory) should diminish performance. Longer feedback processing time should produce larger criterion values if the explicit system is involved in developing base-rate sensitivity.

### Implicit and explicit contributions to base-rate sensitivity

Our results support the conclusion that implicit learning is involved in developing base-rate sensitivity from experience in perceptual classification. In both experiments, criterion values were reliably larger (i.e., closer to optimal) in the response conditions than in the observational conditions. This was true for criterion values derived from signal detection analysis as well as decision bound modeling analysis. Accuracy rates were also higher in the response conditions, as should be expected [8, 33]. Participants in observational conditions barely adjusted their decision criterion values away from an unbiased criterion reflecting insensitivity to base-rates. Modeling analysis, which provides a statistical test for each individual participant, indicated that a significantly greater proportion of data sets in the observational condition than in the response condition were best accounted for by a model assuming an unbiased decision criterion. This was particularly clear for participants in Experiment 2.

Our results also suggest that explicit learning plays a limited role in learning base-rates from experience in perceptual classification tasks. We found no main effect of feedback processing time in any condition (in all cases Bayes Factor values favored the null hypothesis). However, we did find some interesting patterns related to feedback processing time throughout our results.

Fig 4 might give the impression that base-rate sensitivity was slightly greater in long- than in the short-feedback processing conditions in Experiment 1. However, this difference was never close to significant in any block of response training conditions (minimum p-value was .25). In Experiment 2, there were significant effects of feedback processing time in blocks 2 & 3, but the trends were in opposite directions over these blocks. By the end of training (blocks 4 & 5) there were no significant differences based on processing time.

The modeling results indicate some impact of the feedback processing time manipulation in response conditions. In both Experiments 1 and 2, the results indicate participants were more likely to use an unbiased decision criterion in the *long* feedback processing condition (collapsed across blocks: Experiment 1 *z* = 3.43, *p* < .01; Experiment 2 *z* = 2.34, *p* < .01). This pattern of results may seem counterintuitive, but makes sense in the context of earlier research into COVIS predictions. Several other studies have shown that disruption of processing in the explicit system may actually *help* the implicit system function better [32, 44, 45].

The modeling and signal detection results together constitute an additional manipulation check on the feedback processing-time manipulation. The modeling results from both experiments suggest that the delay manipulation was sufficient to affect performance in response conditions (but not observational conditions). In the response conditions—in which implicit learning was engaged—longer feedback processing time led more participants to fail to adjust their criterion at all (indicated by the higher percentage of subjects best fit by the unbiased model).

It also appears that the baseline training in Experiment 1 may have interfered with criterion adjustment on subsequent unequal base-rate trials. Experiment 2 indicates that participants can begin showing base-rate sensitivity early in training. This interference only affected performance in response training conditions. Given that the baseline phase involves response training (i.e., engages the implicit system which learns associatively over time), it makes sense that the unbiased criterion learned in that phase may be resistant to rapid change and require significant re-training before change is evident. Notably, removal of this impediment in Experiment 2 did nothing to increase base-rate sensitivity in the observational training conditions, in early blocks or otherwise.

One could argue that a more direct test of the explicit system’s contribution to base-rate learning would be to provide learners with a summary of base-rates throughout classification training. This seems unlikely to help, though, given the literature showing the limits of summary information on base-rate sensitivity. However, it makes sense to examine the extent to which summary information combines with direct experience to help—or perhaps even hinder—base-rate sensitivity. If the explicit learning system does not significantly contribute when the implicit system is engaged (as we conclude from our response training conditions) then adding summary base-rate information likely will not improve sensitivity either. It would be particularly interesting to see if summary information aids learning in observational conditions when the implicit learning system is disrupted but the explicit system remains intact. We are conducting this research currently.

An alternative account of our results could be that differences existed in motivation level between response and observational conditions; participants might simply have been bored in observational conditions. There are several reasons to doubt this explanation. First, response accuracy in the secondary task was high in both response and observational training conditions, suggesting that participants were engaged across blocks of trials. Second, in recent work [17] we disrupted implicit learning by delaying classification feedback (participants responded on each trial) and again found larger criterion values when implicit learning was supported. Third, in previous studies comparing response and observational training using 2-dimensional categories, observational training performance was undiminished relative to response training in conditions where explicit learning should succeed. In these studies, if participants were less motivated during observational training, it did not affect performance (e.g., [25, 26]). Finally—and perhaps most compelling—the results of our Experiment 2 show clear differences between criterion placement in response and observational conditions as early as the first block of trials. And there were no baseline trials in this experiment. It seems unlikely that observational learning participants had lower motivation from the very beginning of training blocks.

### Links to neuroscience of categorization

The studies reported here are important for several reasons. A great deal of time and effort has gone into understanding acquisition and use of base-rate knowledge, reflecting the importance of this information to decision making. Most of this research focuses on base-rate summaries provided for explicit reasoning. Our results suggest that engaging the appropriate learning system may be important for base-rate sensitivity in classification judgments. And although some have speculated that base-rate sensitivity from experience entails implicit learning, this assumption has received little empirical testing (although see [17]). This is important because the many classification studies showing base-rate sensitivity gained from experience do not necessarily indicate implicit learning. However, regarding sensitivity to base-rates through direct-experience classification, our results do support the assumption that implicit learning is involved.

Furthermore, the current work underscores the relevance of base-rate influence to cognitive neuroscience research into category learning. The present studies were motivated by the observation that category learning from experience may or may not involve implicit learning. We must point out that, although the assumptions of COVIS suggest that base-rate sensitivity might rely on basal-ganglia mediated implicit learning, the theory does not specifically make this prediction; its predictions focus on dimensional rule learning. However, more recent work in this area makes a distinction between category rule learning and criterion learning. With 2-dimensional category structures, the participant must determine which dimension(s) to include in the categorization rule (i.e., rule learning). Once that is determined, the learner must still discover the most optimal placement of the decision criterion. In the case of our 1-dimensional stimuli, the rule itself would be considered a verbalizable rule; the domain of the explicit learning system. However, our results suggest that criterion placement itself may require a nonverbalizable implicit learning process, particularly in the case of unequal category base-rates. In this regard, the current results correspond to recent computational neuroscience work arguing for associative learning of criterion placement [43]. It is clear from our current results that participants were limited in their criterion adjustment in response to base-rates when implicit learning was disrupted.
